# Recurrence and Disease-Free Survival in Head and Neck Squamous Cell Carcinoma After Margin-Free Resection on Frozen Section: An Institutional Perspective

**DOI:** 10.7759/cureus.11385

**Published:** 2020-11-08

**Authors:** Atif A Hashmi, Syeda N Iftikhar, Rimsha Haider, Nabeel N Baig, Muhammad Ghani Asif, Muhammad Irfan

**Affiliations:** 1 Pathology, Liaquat National Hospital and Medical College, Karachi, PAK; 2 Internal Medicine, Liaquat National Hospital and Medical College, Karachi, PAK; 3 Emergency Medicine, National Institute of Blood Diseases and Bone Marrow Transplantation, Karachi, PAK; 4 Research and Development, College of Physicians and Surgeons, Karachi, PAK; 5 Pathology, Multan Medical and Dental College, Multan, PAK; 6 Statistics, Liaquat National Hospital and Medical College, Karachi, PAK

**Keywords:** head and neck squamous cell carcinoma (hnscc), oral squamous cell carcinoma, frozen section, recurrence, disease-free survival, margin-free resection, gutka, areca nut, human papilloma virus (hpv)

## Abstract

Introduction

The most important factor determining survival in patients with head and neck squamous cell carcinoma (HNSCC) is a disease recurrence. A high rate of recurrence was noted in previous studies conducted in Pakistan; however, these studies did not consider margin status as inadequate margin clearance leads to disease recurrence. In this study, we determined cancer recurrence in patients with HNSCC after nullifying this factor.

Methods

This cross-sectional observational study was conducted in Liaquat National Hospital (LNH) for a duration of three years. Data collection period was from January 2015 to December 2017. A total of 150 patients that underwent surgery at LNH for HNSCC with margin-free frozen sections were included in the study. Pathological tumor characteristics such as tumor type, size, depth of invasion and nodal status were determined.

Results

The mean age of the patients was 50.31±12.90 with mean tumor size of 3.38±1.76. Nodal metastases were present in 45.3% cases with 17.3% showing extranodal extension. Recurrence was observed in 66% of cases with median disease-free survival of 12 months and perineural invasion was noted in 12% cases. We found a significant association of disease recurrence with larger tumor size, depth of invasion and extranodal extension. Moreover, younger age (<30 years) and older age (>50 years) groups showed higher rates of recurrence than the middle age group (30-50 years). Similarly, univariate and multivariate analyses revealed that tumors with ≥1 cm depth of invasion and the presence of extranodal extension were more likely to have disease recurrence than tumors with <1 cm depth of invasion and without extranodal extension. Survival analysis using the Kaplan-Meier method for HNSCC revealed a significant difference in disease-free survival in patients with more than 2 cm tumor size and ≥1 cm depth of invasion than cases with ≤ 2cm tumor size and <1 cm depth of invasion.

Conclusion

A high rate of disease recurrence for HNSSC was noted in our study, despite margin-free primary tumor resection. Apart from tumor size and depth of invasion, extranodal extension was significantly associated with disease recurrence in HNSCC. This signifies a need for margin evaluation of neck dissection specimen in cases with extranodal extension.

## Introduction

Our region (South-east Asia) is considered a high-risk area for head and neck squamous cell carcinoma (HNSCC) owing to the wide use of areca-nut chewing in the form of gutka and pan [1-3]. It was proposed that areca-nut induced HNCC is more prevalent than human papilloma virus (HPV)-induced HNSCC in this region [4]. These cancers are considered more aggressive than HPV-induced oral cancers in western countries. Alternatively, a low frequency of HPV-induced HNSCC was depicted in a few studies conducted in Pakistan [5,6].

The most important factor determining survival in patients with HNSCC is disease recurrence. A high rate of recurrence was noted in previous studies conducted in Pakistan [7]. Whether this high rate of recurrence is secondary to inadequate margin clearance or not is unknown as margin-free resection is the most important factor determining recurrence in most cancers. Most of the previous studies that evaluated recurrence in HNSCC in our population did not consider margin status as inadequate margin clearance leads to disease recurrence, and thus is an important confounding factor. In this study, we determined cancer recurrence in patients with HNSCC after nullifying this factor.

## Materials and methods

**Figure 1:**
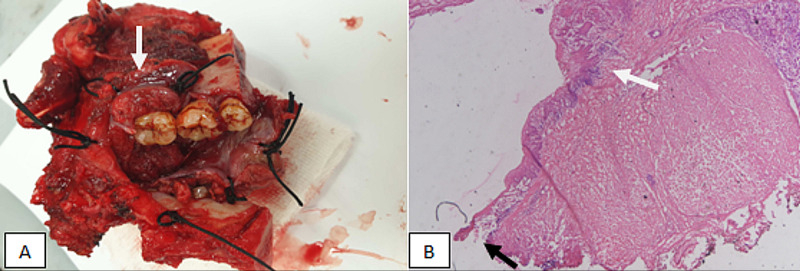
(A) Gross specimen of oral cancer specimen sent for frozen section, tumor present on buccal mucosa (white arrow), and margins are marked with sutures. (B) Microscopic frozen sections showing tumor (white arrow) more than 1 mm from the resection margin (black arrow).

This cross-sectional observational study was conducted in Liaquat National Hospital (LNH) for a duration of three years. Data collection period was from January 2015 to December 2017. Patients that underwent surgery at LNH for HNSCC with margin-free frozen sections were included in the study. More than 1 mm tumor-free margin on the frozen section was taken as tumor-free resection margin (Figure 1).

All the cases included in the study had the diagnosis of squamous cell carcinoma on incisional biopsy before definitive surgical resection. All these patients presented in the Otorhinology clinics of LNH with head and neck mass or ulcerated lesions. After clinical examination and further workup, including computed tomography (CT) scans, incisional biopsy of the lesion was taken. Cases with post-neoadjuvant chemotherapy or radiation were excluded from the study along with patients having any evidence of systemic metastasis. Any patient with the positive margin on frozen section was also excluded from the study. Tumor present on resected margin or less than 1 mm distance of the tumor from the resected margin was considered a positive margin on the frozen section. Any case with positive margin on the final (paraffin) section was also excluded from the study. After approval from the research and ethical committee of the hospital, pathological and oncological records were evaluated and data were entered. Frozen section reports were assessed along with final histopathology reports of all patients. Pathological tumor characteristics such as tumor type, size, depth of invasion and nodal status were noted. The disease-free survival was determined by reviewing the oncological records.

All specimens (whole resected specimen) were received fresh for frozen section examination. After evaluation of size and dimensions of the specimen, the tumor was located and size and extent of the tumor were assessed. Gross distances of the tumor from all the marked (by the surgeon) surgical resection margins were measured. All margins were inked with different colored inks. If the resected margin was more than 1 cm from the tumor, then shaved sections were taken. Alternatively, if the gross distance of the tumor from the resected margin was less than 1 cm, multiple radial sections were taken from the margin to measure the distance of the tumor from the margin microscopically. After frozen section reporting, specimens were put into the formalin-filled containers and kept for 24 h for fixation. After 24 h, specimens were examined again and additional sections were taken from the tumor to assess tumor differentiation, perineural invasion and depth of invasion. Moreover, sections that were submitted for frozen sections were again assessed microscopically after formalin fixation to note any discrepancy from frozen section reports.

Data analysis was performed using Statistical Package for Social Sciences (Version 26.0, IBM Inc., Armonk, NY). Chi-square and Fisher exact tests were used to check the association. The odds ratio was computed using univariate logistic regression. The multivariate binary logistic regression was applied for variables that were significant on univariate logistic regression. Survival analysis was done by the Kaplan-Meier method. P-values ≤ 0.05 were considered as significant.

## Results

Clinicopathologic features of population under study

A total of 150 cases of HNSCC, were included in the study. The mean age of the patients was 50.31±12.90 with mean tumor size of 3.38±1.76. Most of the tumors were between 2 and 4 cm tumor sizes (49.3%), and 42.7% cases had a depth of invasion ≥ 1 cm. Majority of the patients were male (75.3%) with the oral cavity being the most common site of the tumor (69.3%). Nodal metastases were present in 45.3% cases with 17.3% showing extranodal extension. Most of the tumors were keratinizing (60%) and 62.7% were grade II (moderately differentiated). Recurrence was observed in 66% of cases with median disease-free survival of 12 months and perineural invasion was noted in 12% cases (Table 1).

**Table 1:** Clinicopathologic features of population under study SD, standard deviation

Clinicopathological characteristic	Frequency (%)
Age (years)	
Mean ± SD	50.31±12.90
Age groups	
≤30 years	17(11.3)
31–50 years	68(45.3)
>50 years	65(43.3)
Tumor size (cm)	
Mean ± SD	3.38±1.76
Tumor size groups	
≤2 cm	32(21.3)
2.1–4 cm	74(49.3)
>4 cm	44(29.3)
Depth of invasion (cm)	
Mean ± SD	1.08±0.68
Depth of invasion groups	
<1 cm	79(52.7)
≥1 cm	71(47.3)
Disease-free survival (months)	
Mean± SD	25.27±22.51
Median	12.00
Gender	
Male	113(75.3)
Female	37(24.7)
Site of tumor	
Oral cavity	104(69.3)
Lip	2(1.3)
Tongue	36(24)
Soft palate	8(5.3)
Nodal metastasis	
Present	68(45.3)
Absent	82(54.7)
Extranodal extension	
Present	26(17.3)
Absent	124(82.7)
Histological subtypes	
Non-keratinizing	14(9.3)
Keratinizing	90(60)
Non-keratinizing with maturation	46(30.7)
Histological grade	
Grade-I	42(28)
Grade-II	94(62.7)
Grade-III	14(9.3)
Perineural invasion	
Present	18(12)
Absent	132(88)
Recurrence	
Yes	99(66)
No	51(34)

Among 99 cases in which recurrence was observed, 61 cases (61.6%) had recurrence at the primary tumor site and 38 cases (38.3%) had recurrence in the neck. 

The most common risk factor was pan/ gutka usage (62.0%) followed by smoking (10.7%). Adjuvant chemotherapy and radiation therapy were given in 57.3% and 59.3% cases, respectively (Table 2).

**Table 2:** Frequency of risk factors and adjuvant therapy in patients with head and neck squamous cell carcinoma included in the study (n = 150)

Risk factor	Frequency (%)
History of Pan/ gutka usage	
Present	93 (62.0%)
Absent	57 (38.0%)
Smoking history	
Present	16 (10.7%)
Absent	134 (89.3%)
Alcohol history	
Present	3 (2.0%)
Absent	147 (98.0%)
Adjuvant therapy	
Chemotherapy	
Given	86 (57.3%)
Not given	64 (42.6%)
Radiation	
Given	89 (59.3%)
Not given	61 (40.7%)

Association of clinicopathologic features with disease recurrence

Table 3 shows the association of clinicopathologic features with disease recurrence. We found a significant association of disease recurrence with larger tumor size, depth of invasion and extranodal extension. Moreover, younger age (<30 years) and older age (>50 years) groups showed a higher rate of recurrence compared to the middle age group (30-50 years). No significant association of disease recurrence with any other clinicopathological characteristic was noted in our study.

**Table 3:** Association of clinicopathological features with disease recurrence *Chi-square test was applied, **Fisher exact test was applied.

Clinicopathological characteristics	Recurrence		P-value
	n (%)		
	Yes	No	
Gender			
Male	72(72.7)	41(80.4)	0.302*
Female	27(27.3)	10(19.6)	
Age group			
≤30 years	15(15.2)	2(3.9)	0.043*
31–50 years	39(39.4)	29(56.9)	
>50 years	45(45.5)	20(39.2)	
Tumor size			
≤2 cm	12(12.1)	20(39.2)	<0.0001*
2.1–4 cm	51(51.5)	23(45.1)	
>4 cm	36(36.4)	8(15.7)	
Depth of invasion			
<1 cm	42(42.4)	37(72.5)	<0.0001*
≥1 cm	57(57.6)	14(27.5)	
Site			
Oral cavity	69(69.7)	35(68.6)	0.326**
Lip	0(0)	2(3.9)	
Tongue	24(24.2)	12(23.5)	
Soft palate	6(6.1)	2(3.9)	
Nodal metastasis			
Present	42(42.4)	26(51)	0.319*
Absent	57(57.6)	25(49)	
Extra nodal extension			
Present	12(12.1)	14(27.5)	0.019*
Absent	87(87.9)	37(72.5)	
Histological subtypes			
Non-keratinizing	12(12.1)	2(3.9)	0.107**
Keratinizing	54(54.5)	36(70.6)	
Non-keratinizing with maturation	33(33.3)	13(25.5)	
Histological grade			
Grade-I	30(30.3)	12(23.5)	0.131**
Grade-II	57(57.6)	37(72.5)	
Grade-III	12(12.1)	2(3.9)	
Perineural invasion			
Present	12(12.1)	6(11.8)	0.949*
Absent	87(87.9)	45(88.2)	

Similarly, univariate and multivariate analyses revealed that tumors with ≥1 cm depth of invasion and the presence of extranodal extension were more likely to have disease recurrence than tumors with <1 cm depth of invasion and without extranodal extension (Table 4).

**Table 4:** Univariate and multivariate analysis for disease recurrence with clinicopathological features of population under study Univariate and multivariate binary logistic regression were applied. *Reference group. CI, confidence interval.

Clinicopathological characteristics	P-value	Odds ratio (95% CI)	P-value	Adjusted Odds ratio (95% CI)
Gender				
Male	0.304	0.650(0.286-1.478)		
Female*		1		
Age groups				
≤30 years	0.132	3.333(0.696-15.968)		
31–50 years	0.157	0.598(0.293-1.219)		
>50 years*		1		
Tumor size				
≤2 cm	<0.0001	0.133(0.04-0.380)		
2.1–4 cm	0.128	0.493(0.198-1.225)		
>4 cm*		1		
Depth of invasion				
<1 cm	0.001	0.279(0.134-0.580)	0.001	0.276(0.131-0.584)
≥1 cm*		1		1
Site				
Oral cavity	0.618	0.657(0.126-3.426)		
Lip	0.999	0.000(0.000-0.000)		
Tongue	0.649	0.667(0.117-3.813)		
Soft palate*		1		
Nodal metastasis				
Present	0.319	0.709(0.360-1.396)		
Absent*		1		
Extranodal extension				
Present	0.022	0.365(0.154-0.863)	0.027	0.359(0.145-0.887)
Absent*		1		
Histological subtypes				
Non-keratinizing	0.301	2.364(0.464-12.048)		
Keratinizing	0.179	0.591(0.274-1.274)		
Non-keratinizing with maturation*		1		
Histological grade				
Grade-I	0.295	0.417(0.081-2.148)		
Grade-II	0.086	0.257(0.054-1.213)		
Grade-III*		1		
Perineural invasion				
Present	0.949	0.967(0.340-2.746)		
Absent*		1		

**Figure 2:**
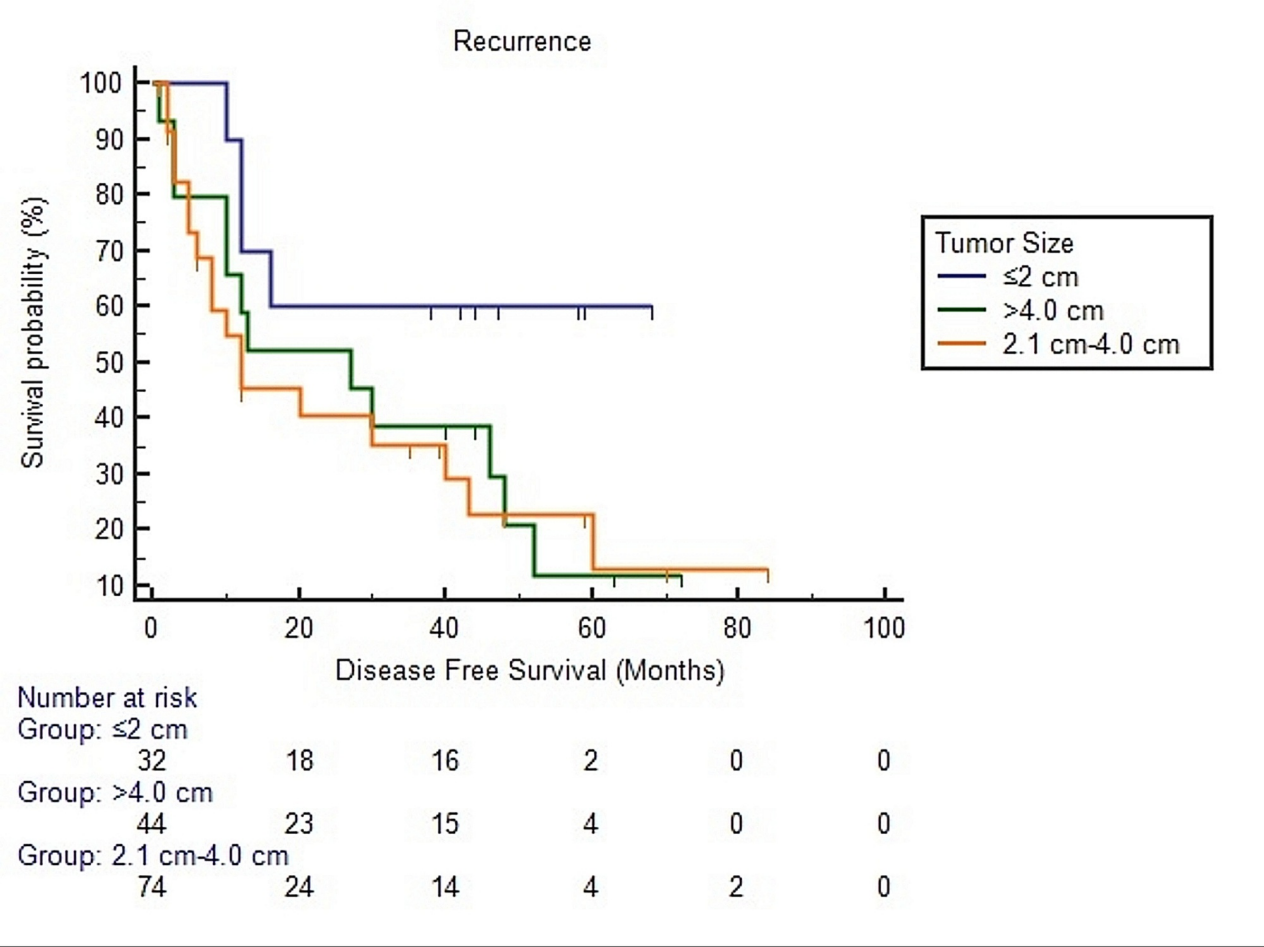
Survival analysis using the Kaplan-Meier method for head and neck squamous cell carcinoma with respect to tumor size Log rank p-value = 0.002

**Figure 3:**
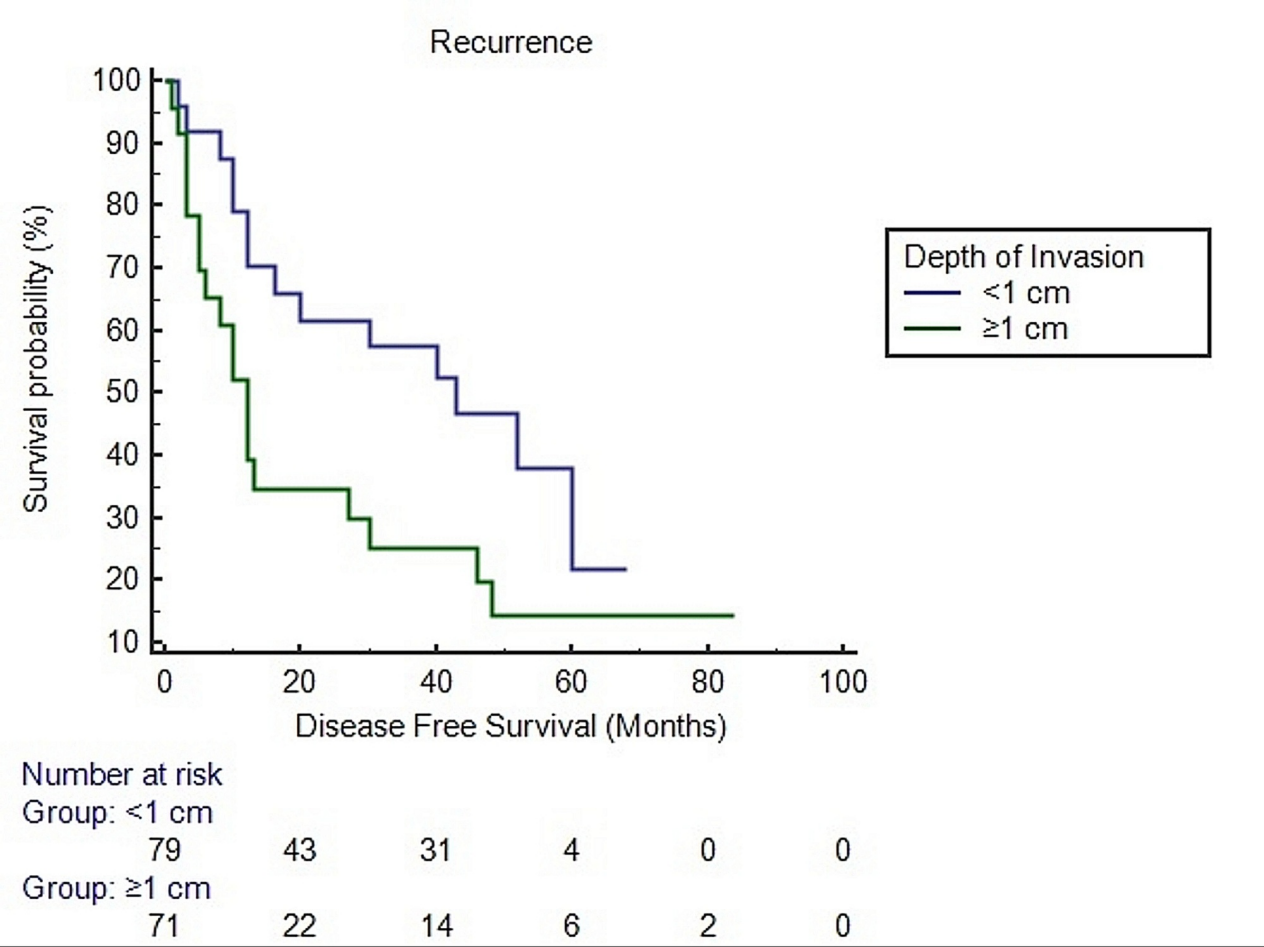
Survival analysis using the Kaplan-Meier method for head and neck squamous cell carcinoma with respect to depth of invasion Log rank p-value = <0.0001

**Figure 4:**
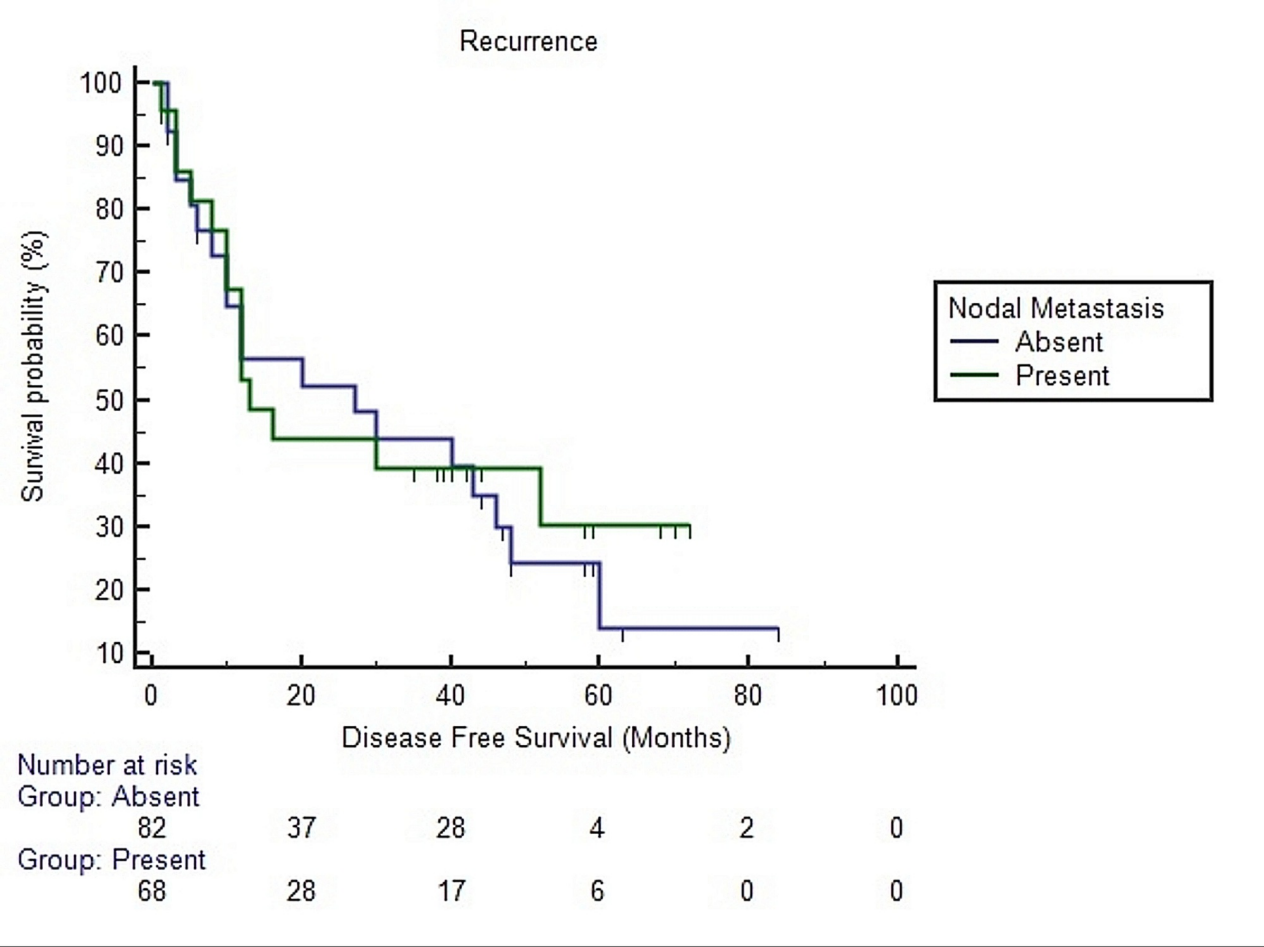
Survival analysis using the Kaplan-Meier method for head and neck squamous cell carcinoma with respect to nodal metastasis Log rank p-value = 0.526

Survival analysis using the Kaplan-Meier method for HNSCC revealed a significant difference in disease free survival in patients with more than 2 cm tumor size and ≥1 cm depth of invasion than cases with ≤2 cm tumor size and <1 cm depth of invasion (Figures 2, 3, 4).

## Discussion

In this study, we found a high rate of recurrence in our cases of HNSCC despite tumor-free resection margins on the frozen section. Moreover, we noted that the presence of recurrence was significantly associated with tumor size, depth of invasion and extranodal extension.

Various studies have evaluated the recurrence of HNSCC in locoregional population. A study conducted in China assessed disease recurrence in 275 patients with oral squamous cell carcinoma. They reported 32.7% recurrence rate in their study; however, in their study, 66.2% cases were early-stage cancers (T1+T2). Recurrence in late-stage cancers (T3+T4) in their study was 57%. They also found that tumor (T) stage, nodal (N) stage and degree of differentiation were the major factors determining disease recurrence [8]. We also found a significant association of tumor size and depth of invasion with disease recurrence; however, no significant association of disease recurrence was noted with the degree of differentiation in our study. Another study, conducted in Brazil, compared cases of HNSCC with and without recurrence. They found that the site (tongue) and degree of differentiation were the factors associated with disease recurrence [9]. However, we did not find any significant association of tumor site with disease recurrence in our study. An important factor that was not assessed carefully in these studies was extranodal extension as a recurrence of HNSCC typically occurs either at the primary site or in the neck and presence of extranodal extension is an important factor that can lead to disease recurrence in the neck. Although margins of primary tumor are carefully assessed and closed margins are re-shaved to get a margin-free resection, neck specimen is typically not assessed for margins and therefore, in the presence of extranodal extension, the chances of recurrence in the neck increase.

An important prognostic factor in oral cancers is margin-free resection, and the frozen section has pivotal importance in this regard. There are two ways for intra-operative pathological consultation. The first and more prevalent is tumor-bed margin evaluation and second is sending a whole resected specimen for frozen section assessment (tumor-specific margin). In tumor-bed evaluation for the frozen section, the surgeon after removal of tumor sends shaving from resected tumor bed. Any tumor present in the shaving specimen is considered positive and re-shaving is done. The disadvantage of this technique is that the differentiation between a positive margin and close margin (i.e. <5 mm) is not possible. Second, in case of large tumors, tumor bed is huge and evaluation of whole tumor bed is impossible. The second method, i.e. evaluation of whole specimen by frozen section, although cumbersome and time-consuming has recently been proved to be more effective in reducing the chances of recurrence and improving disease-free survival in patients with oral squamous cell carcinoma [10-12]. We at LNH routinely practice this technique, in which all the margins of the resection specimen are evaluated on frozen section and rapidly communicated to the surgeon. Despite margin-free resections, we found a high rate of recurrence in our study. This signifies the importance of other factors that affect disease recurrence, that is, tumor size and depth of invasion. Moreover, the poor prognostic importance of extranodal extension should not be underscored in these circumstances.

One of the major limitations of our study was limited sample size. Second, molecular analysis for HPV status was not determined, as HPV-induced HNSCC behave in a less aggressive fashion than non-HPV induced cancers. Although we found significant associations in our study, there were multiple confounding factors that could not be controlled in such study design, for instance, different combinations of chemotherapy and radiation therapies were given to different patients based on oncologist decision and multidisciplinary team meetings that could also alter individual outcomes. 

## Conclusions

A high rate of disease recurrence was noted in HNSCC in our study after tumor-free resection margins on the frozen section. A substantial proportion of recurrence occurred in the neck in our study. Apart from tumor size and depth of invasion, which are known risk factors of disease recurrence in HNSCC, we also noted a significant association of disease recurrence with extranodal extension. Therefore, we suggest that, in the presence of extranodal extension, resection margins of neck dissection specimen should also be assessed along with primary specimen resection margins.
